# A Time-Varying Effect Model (TVEM) of the Complex Association of Tobacco Use and Smoke Exposure on Mean Telomere Length: Differences between Racial and Ethnic Groups Assessed in the National Health and Nutrition Examination Survey

**DOI:** 10.3390/ijerph191711069

**Published:** 2022-09-04

**Authors:** Francisco Alejandro Montiel Ishino, Claire E. Rowan, Kevin Villalobos, Janani Rajbhandari-Thapa, Faustine Williams

**Affiliations:** 1 Division of Intramural Research, National Institute on Minority Health and Health Disparities, National Institutes of Health, Bethesda, MD 20892, USA; 2 Department of Epidemiology, Rollins School of Public Health, Emory University, Atlanta, GA 30322, USA; 3 Department of Health Policy and Management, College of Public Health, University of Georgia, Athens, GA 30602, USA

**Keywords:** telomere, cotinine, tobacco use, environmental tobacco smoke, time-varying effect modeling (TVEM), health disparity, minority and vulnerable populations

## Abstract

Telomere length is affected by lifestyle and environmental factors and varies between racial and ethnic groups; however, studies are limited, with mixed findings. This study examined the effects of tobacco use and smoke exposure on mean telomere length to identify critical age periods by race/ethnicity. We used time-varying effect modeling on the National Health and Nutrition Examination Survey for continuous years 1999–2002 to observe the effects of active tobacco use and environmental tobacco smoke—measured through serum cotinine—and mean telomere length for adults 19 to 85 and older (N = 7826). Models were run for Mexican American, other Hispanic, non-Hispanic White, non-Hispanic Black, and other/multi-race categories to allow for time-varying group differences, and controlled for biological sex, socioeconomic status, education, and ever-smoker status. Serum cotinine was found to have an increasing effect on telomere length from age 37 to approximately age 74 among Mexican Americans. Among other/multi-race individuals serum cotinine was found to have a decreasing effect at approximately age 42, and among Blacks, it had an overall decreasing effect from age 61 to 78. Findings reveal a further need to focus additional support and resources to intervene regarding disparate health effects from tobacco use and environmental smoke exposure for already vulnerable groups at particular ages.

## 1. Introduction

Telomeres are the DNA-protein sequences that cap and subsequently protect chromosomes [1]. Telomere length shortens gradually with age, and is inversely associated with oxidative stress, inflammation, and cellular senescence; thus, telomere length (TL) is proposed as a biomarker for biological aging [2,3]. Meta-analyses of prospective studies provide evidence that TL is inversely associated with chronic diseases such as cardiovascular disease [4], diabetes [5], and all-cause mortality [6,7]. The literature also suggests that TL varies with demographic and lifestyle characteristics. For instance, there is consensus between studies that TL decreases with increasing age, and that TL is shorter in males compared to females [8,9]. The literature suggests variation in TL according to race/ethnicity, but there is a lack of consensus between studies; compared to non-Hispanic Whites, some studies report a longer TL in African Americans and Hispanics [10,11,12,13,14], whereas others report a shorter TL in these ethnic groups [15,16]. Similarly, inconsistency in TL varies according to smoking behavior. Although individual studies provide inconsistent findings, two recent meta-analyses suggest that TL is shorter in smokers than non-smokers [17,18]. Specifically, current smokers had shorter TL than former smokers or people who had never smoked [17].

Furthermore, while environmental tobacco exposures studies may incorporate cigarette smoking and related behaviors, which may be associated with multiple negative health outcomes, the combination is not a comprehensive measure of tobacco use and environmental tobacco exposure (hereafter total tobacco exposure) [19,20]. The serum cotinine biomarker can provide a reliable measure to assess smoking behaviors as well as environmental tobacco exposures, but only a few studies have examined TL and cotinine levels among adults [21,22,23]. A previous study, using the National Health and Nutrition Examination Survey (NHANES), examined smoking (number of cigarettes per day) as well as serum cotinine levels by race/ethnicity, and found shorter TL to be associated with increased cotinine concentration among Whites, but not among Blacks, and with increased cigarette consumption among both Blacks and Whites [22].

A comprehensive understanding of TL and smoking behavior has been limited primarily to multiple regression approaches estimating the associations of exposure variables (i.e., smoking or tobacco exposure) with telomere attrition [22,24]. However, a recent meta-analysis of 18 longitudinal cohorts [18] tested this causation hypothesis and found no evidence of significantly faster telomere attrition in smokers. Notably, the same meta-analysis found that smokers had shorter TL than non-smokers at all measured time-points, but there was no evidence that this cross-sectional difference increased with age. These findings suggest the importance of timing effects on the relationship between smoking and TL, which is not addressed by most linear models. The association between tobacco use and environmental tobacco smoke may also change with time, as various factors can affect use and exposure patterns. Furthermore, given the limited availability of longitudinal cohort data, the association of change over time is unclear, especially between racial and ethnic groups. Our purpose was to examine the effects of tobacco exposure on mean telomere length by race/ethnicity across age. More specifically, we explored the time-varying relationship of total tobacco exposure, measured by serum cotinine, on mean telomere length between Mexican Americans, other Hispanics, non-Hispanic Whites, non-Hispanic Blacks, and other/multi-racial groups. We expected variation in the effect of tobacco exposure on mean telomere length across age by race/ethnicity.

## 2. Materials and Methods

We examined changes in mean TL length across age by using participants’ ages as a proxy for a longitudinal measure of TL across time. We compared the nature of the time-varying relation between TL and total nicotine exposure (serum cotinine levels ≥10 ng/mL) among different racial/ethnic groups. There is a gap in the literature regarding the association of total tobacco exposure (active tobacco use and environmental tobacco smoke exposure) with TL, particularly regarding differences in association by race and ethnicity. This may be an artefact of multiple regression approaches having been used to estimate exposures and the rate of TL attrition to capture transitional or latent time-varying effects [22,24]. Current evidence makes assessing time’s effects on the relationship between smoking and TL plausible; however, to expand the literature, multiple longitudinal cohorts would be necessary.

### 2.1. National Health and Nutrition Examination Survey (NHANES) Data

We used data from the NHANES 1999–2002 survey cycles. Data used for this analysis are publicly available at the US Centers for Disease Control and Prevention—National Center for Health Statistics—NHANES repository (https://www.cdc.gov/nchs/nhanes/index.htm, accesses on 12 March 2019). Briefly, the NHANES is a nationally representative survey of non-institutionalized US populations. The methodological details for the 1999–2002 NHANES are defined elsewhere [25]. Our analysis was limited to 7827 individuals with telomere length assessment. There were 4260 participants in 2001–2002 and 3567 in 1999–2000. One observation was omitted due to the mean telomere length being 350% over the sample mean. Given the complex survey design, our analyses were weighted to account for the 1999 to 2002 cycles. Data used for our analysis are available upon reasonable request.

### 2.2. Measures

#### 2.2.1. Telomeres

Blood samples were obtained from NHANES survey participants, which were stored to conduct later analyses on DNA samples. Telomeres were collected from leukocytes. Telomere assays to assess TL relative to standard reference DNA (T/S ratio) were performed at the University of California, San Francisco, under the direction of Dr. Elizabeth Blackburn, using a quantitative polymerase chain reaction; the procedure and full process description can be found in detail elsewhere [10,26]. Human telomere length can range from 5 to 15 kb (i.e., approximately 0.879 to 2.638 T/S ratio); the length varies by cell type, as well as other social, environmental, and genetic factors [27,28,29,30].

#### 2.2.2. Serum Cotinine

Cotinine, a major metabolite of nicotine, is measured in serum in NHANES and is used as a marker for both active and passive smoking or exposure to environmental tobacco smoke. Briefly, cotinine concentrations (ng/mL) were derived using an isotope dilution high-performance liquid chromatography technique. The detailed laboratory methodology for serum cotinine assessment is described elsewhere [26,31].

#### 2.2.3. Age

We used age as a continuous time scale that included adult participants aged 19 to 85 and older. The oldest NHANES 1999–2002 continuous cycle participants were grouped as age 85 and older due to disclosure concerns [25].

#### 2.2.4. Race/Ethnicity

Each model was assessed using the domain of racial and ethnic identity that was self-identified by NHANES participants. Race/ethnicity categories included Mexican American, other Hispanic, non-Hispanic White, non-Hispanic Black, and other race/multi-racial.

#### 2.2.5. Controls and Covariates

Sex (male and female), education (below high school and high school or above), socio-economic status (SES), and lifetime cigarette use were used as controls and covariates due to their known effects on telomere length [10,14,16,17]. SES was measured in terms of the poverty-income-ratio (PIR), which is the ratio of family income to the poverty threshold; PIR was calculated by dividing family income by the number of family members, using US Health and Human Services poverty guidelines by state and fiscal year [25,32]. A PIR of 1 indicated the official federal poverty threshold; participants were classified as above poverty level (PIR > 1) and at or below poverty level (PIR ≤ 1). Covariates included smoking behavior as a lifetime cigarette smoker or ever-smoker (yes/no) based on if the participant had consumed more than 100 cigarettes in their lifetime.

### 2.3. Data Analysis

Time-varying effect modeling (TVEM) is a decades old nonparametric model, requiring no constraints on shapes of intercept and slope functions [33]. Strength of association and directionality are evaluated along the continuum of a time scale, and for our purposes used the age of multiple NHANES participants to ensure adequate coverage. TVEM provided us the flexibility to measure time as age on a continuous scale with parameter estimates that changed with time [33,34,35,36]. To identify the temporal relations of age to serum cotinine and telomere length, we fit the following model to the 1999–2002 NHANES cycles by race/ethnicity:

*TelomereLength_ij_* = *β*_0_(*t*) + *β*_1_(*t*)∗*SerumCotinine_ij_* + *β*_2_(*t*)∗*Sex_ij_* + *β*_3_(*t*)∗*Education_ij_* + *β*_4_(*t*)∗*SocioeconomicStatus_ij_* + *β*_5_(*t*)∗*EverSmoker_ij_* + *ε_ij_* (1)

Participant *i* measured at time *t* where assessment *j* was sampled at different times across individuals. Our study used age as a proxy for time. In our equation *β*_0_ (*t*) is the intercept function, which captures an expected telomere length trajectory for a participant with average levels of *SerumCotinine*. The slope function *β*_1_ (*t*) characterizes the gradual temporal projection between the mean telomere changes and *SerumCotinine* over age 20 to 85 and older. We included 4 time-invariant covariates: *β*_2_(*t*) *Sex* [male/female]; *β*_3_(*t*) *Education* [less than high school/at or above high school education]; *β*_4_(*t*) *SocioeconomicStatus* [at or below poverty level using PIR/above poverty level using PIR]; and *β*_5_(*t*) *EverSmoker* status [no/yes]. Random errors *ε_ij_* were assumed to be normally distributed.

The P-spline method was used to estimate and visualize parameter functions. Each function was split into multiple knots (i.e., the splitting points between intervals), and each knot was estimated using a polynomial model (e.g., linear, quadratic, or cubic). As such, TVEM selection was based on a comparative approach using a number of different knots (i.e., we started with six and gradually reduced the number) and evaluated using values from the Akaike and Bayesian information criterion (AIC and BIC, respectively). We then inspected the shapes of estimated functions using linear, quadratic, and cubic models. The model selected for interpretation was based on the lowest AIC and BIC using the cubic model. The *%WeightedTVEM* (version 2.6) macro for SAS [36], which can be downloaded from http://methodology.psu.edu (accessed 24 February 2018), was used to fit the model interpreted. The *%WeightedTVEM* macro accommodated weights and clusters from the NHANES complex survey design. Analytic steps and syntax are available upon request.

## 3. Results

The mean age of the adult sample was 46.1; age ranged from 19 to 85 and older. The sample was almost evenly distributed between males and females, and was predominantly non-Hispanic White (72.9%), with a high school degree/equivalent or above, and living above poverty level. The sample was almost equally classified as either a lifetime non-cigarette smoker or a cigarette smoker. See Table 1 for full details. Supplementary Table S1 provides descriptions by race/ethnicity.

### 3.1. Intercept Function of Telomere Length over Age for Each Racial/Ethnic Group

**Figure 1:**
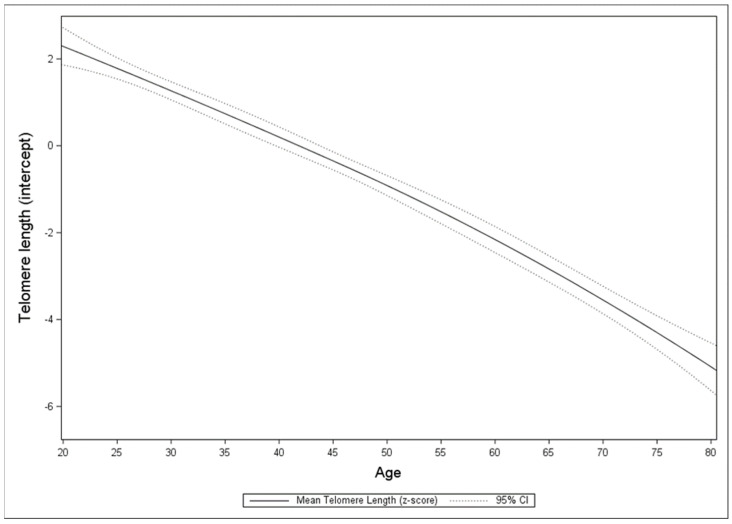
Mean telomere length intercept only model.

**Figure 2:**
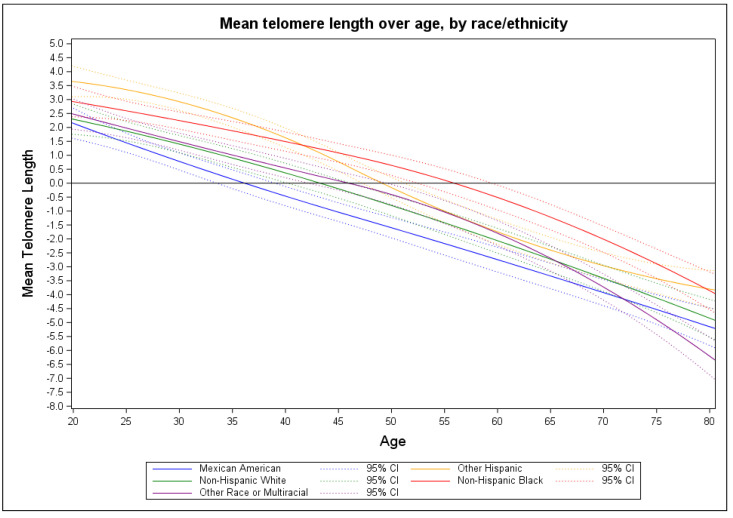
Mean telomere length intercept only models by race/ethnicity.

Figure 1 provides a graphical summary of the intercept functions for telomere length over time represented by participant age for an average person in the NHANES. Figure 2 graphically represents the telomere length for an average individual belonging to a specific racial/ethnic group (i.e., Mexican American, other Hispanic, non-Hispanic White, non-Hispanic Black, or other race/multi-racial).

**Figure 3:**
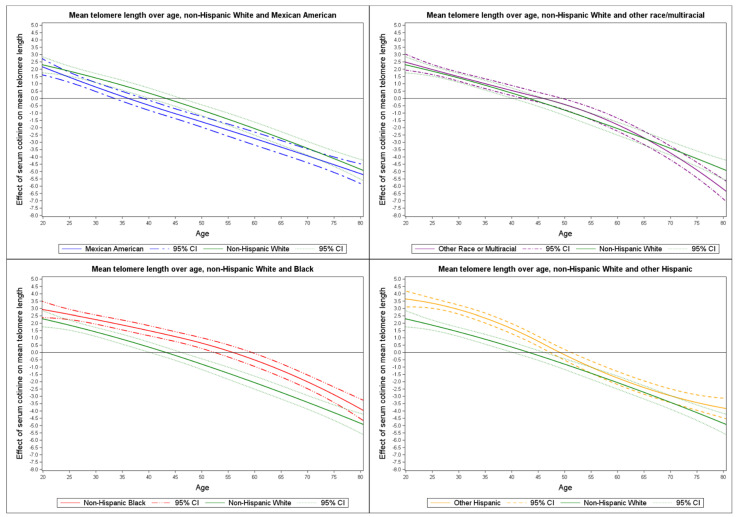
Effect of serum cotinine on mean telomere length by race/ethnicity compared to non-Hispanic White.

Figure 3 provides a graphical summary of the effect of serum cotinine on mean TL for each racial/ethnic group (i.e., Mexican American, other Hispanic, non-Hispanic Black, and other race/multi-racial) compared to non-Hispanic White. We observed a confidence band overlap between Mexican Americans and non-Hispanic Whites with an exception from approximately age 30 to 50 (top left panel). Among other Hispanics we observed a confidence band overlap at approximately age 47. A wider confidence interval band gap occurred from age 25 to 75 between non-Hispanic Blacks and Whites (bottom left panel). The other race/multi-racial and non-Hispanic Whites confidence interval bands overlapped throughout.

### 3.2. Time-Varying Effect Model of Serum Cotinine and Telomere Length over Age by Race/Ethnicity

**Figure 4:**
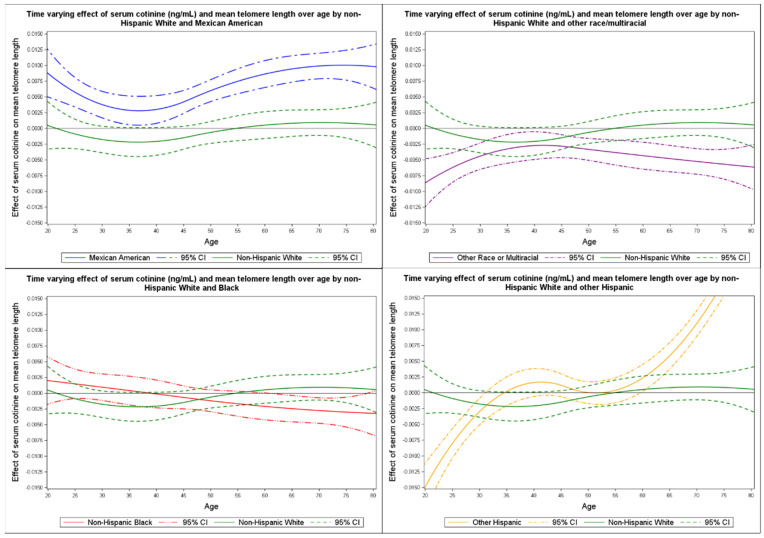
Time varying effect of serum cotinine on mean telomere length by race compared to non-Hispanic Whites.

Slope functions of the effect of serum cotinine on telomere length were similar between Mexican Americans and non-Hispanics; however, confidence bands did not overlap. The greatest mean change in telomere length from active tobacco use and high passive smoke exposure among Mexican Americans occurred at approximately 37 years of age with an increase of 2.81 × 10^−3^ [95% confidence interval (CI): 5.16 × 10^−4^ to 5.10 × 10^−3^] with the highest peak increase occurring at 74.3 years of age by 1.01 × 10^−2^ [95% CI: 7.77 × 10^−3^ to 1.24 × 10^−2^]. The greatest change in telomere length among other race/multi-racial individuals was at age 41.7 with a decrease of −2.68 × 10^−3^ [95% CI: −4.78 × 10^−3^ to −5.57 × 10^−4^], which continued to age 85 and older. Non-Hispanic Blacks were found to have a complex association with serum cotinine, whereby at approximately 61 years of age telomere length decreased until age 78.3 [i.e., −2.18 × 10^−3^ (95% CI: −4.32 × 10^−3^ to −3.66 × 10^−5^) to −3.33 × 10^−3^ (95% CI: −6.16 × 10^−3^ to −1.25 × 10^−4^)]. Non-Hispanic Whites and other Hispanic groups’ telomere length changes could not be confidently reported (see Figure 4).

## 4. Discussion

We used a TVEM to examine racial/ethnic group differences regarding the effects of serum cotinine on telomere length with models run using Mexican American, other Hispanic, non-Hispanic White, non-Hispanic Black, and other/multi-race groups. Models were controlled for biological sex, socioeconomic status, education, and ever-smoker status where we found varied time-varying effects of serum cotinine and telomere length. We found that telomere length increased at age 37 until approximately age 74 among Mexican Americans. In other/multi-race individuals, serum cotinine was found to have a decreasing effect at approximately age 42, and in Blacks, it had an overall decreasing effect from 61 to 78 years of age.

Our findings help fill the current gap in the literature regarding the association of total tobacco exposure (active tobacco use and environmental tobacco smoke exposure) with TL, with special emphasis on race and ethnicity. To overcome the gap in longitudinal data across time, we utilized a TVEM to study the effects of tobacco exposure on TL across age. The TVEM allowed our study to examine time-dependent outcomes and dynamic associations that vary across time [34,35]. Furthermore, we expanded the literature by examining the association of race/ethnicity with TL and nicotine exposure measured through serum cotinine; most current research has not focused on understanding racial/ethnic differences.

Although cotinine is the major nicotine metabolite and is considered a reliable biomarker of tobacco smoke exposure, it is not often used to dynamically understand smoking behaviors and environmental tobacco exposure. Moreover, studies have found that TL variation is related to not only cotinine levels but also to race/ethnicity [37]. For instance, past research has reported significant differences in TL associated with age in Hispanics when compared to Whites [10]. In agreement with prior studies, we found that Mexican Americans had an increase in TL throughout their life course, with two pronounced peaks at ages 37 and 74 years, when accounting for total tobacco exposure. The increase in TL among Mexican Americans was also found to be higher after controlling for confounding from PIR and education. This is important, as increased TL in Mexican Americans has been reported to be associated with higher levels of socioeconomic status and related positive health behaviors [38,39]. Nevertheless, our findings revealed that compared to other TVEMs by race/ethnicity, Mexican Americans’ TL increased when serum cotinine was introduced. This increase may be related to the Hispanic paradox; i.e., while Hispanics may live longer than their White and Black counterparts, their overall quality of life, health risks, and outcomes are worse [38]. Hence the increase in telomere length may be indicative of the telomere length paradox [40]; that is, although a decrease in telomere length could increase cancer risk due to genomic instability, longer telomeres may also increase risk for major cancers [40].

We found complex associations with serum cotinine and TL in the other race/multi-racial and non-Hispanic Black groups. We observed a negative effect of serum cotinine on TL at approximately 42 years of age among other race/multi-racial individuals, with a continual decrease throughout their life course; intercept functions of other race/multi-racial and non-Hispanic Whites overlapped. Among non-Hispanic Blacks, TL was found to decrease from approximately age 61 to 78, but there was no pronounced peak of change by age. This could be explained by shorter life expectancy (e.g., in 2000, in the USA, the life expectancies of Black males and females were 68.2 and 74.9 years, respectively, compared to life expectancies for White males and females, which were 74.8 and 80.0 years, respectively [41]) as well as differences in nicotine and cotinine metabolism rates. Past studies reported racial differences in cotinine metabolism with non-Hispanic Blacks metabolizing cotinine more slowly than non-Hispanic Whites [42,43]. Additionally, non-Hispanic Blacks have been reported to have higher levels of serum cotinine than non-Hispanic Whites [44,45] irrespective of the number of cigarettes smoked. The relationships between TL, race/ethnicity, and total tobacco exposure are complex and not well understood.

### Limitations and Strengths

Our study is among the first to apply a TVEM to understand the effects of total tobacco exposure and TL; however, it had limitations. The NHANES data used were cross-sectional and from continuous years 1999 to 2002. The data’s cross-sectional nature prevented causal inference. However, in the absence of longitudinal data, we examined the 1999–2002 NHANES to model the time-varying effect of serum cotinine and telomere length over age—from a respondent pool that was heterogeneous in age—to explore possible racial/ethnic differences. The 1999–2002 NHANES data were used to this end, as these datasets provided necessary biomarkers, such as telomere length and serum cotinine, that were not available in other complex survey designs or nationally representative datasets.

Despite these limitations, our findings provide an evidence base from which to promote specialized methods and approaches to capture racial/ethnic contexts to improve understanding of dynamic changes occurring among medically underserved and underrepresented groups, while accounting for possible age effects. Our application of a TVEM, in the absence of larger longitudinal cohorts, allowed us to model the effects of time using age as a proxy to further disentangle the effects of tobacco use and exposure by race and ethnicity.

## 5. Conclusions

This study fills a critical gap in our understanding of serum cotinine, as an analog to active tobacco use and environmental tobacco smoke, and telomere length by racial/ethnic groups. We explored racial/ethnic differences in active and passive toxic environmental exposures from tobacco smoke across the life course. Existing studies have mixed findings as to what those differential changes in telomere length may signify as a biomarker of health and disease, which demonstrates a need for diverse and racially/ethnically inclusive cohorts to generalize findings. Future research must include multiple biomarkers of health and disease, and behavioral risk factors to be comprehensive as well as to better model the effects of tobacco exposure on telomere length. Comprehensively modeling environmental effects can help better understand the dynamic effects of tobacco exposures on morbidity and mortality, especially as it translates to medically underserved and underrepresented groups. Our findings and use of time-varying modeling approaches add to the burgeoning public health efforts seeking to mitigate ongoing environmental health disparities while concurrently moving towards health equity.

## Figures and Tables

**Table 1:** Sample sociodemographic variables (N = 7826).

	N	%		
Race/Ethnicity				
Mexican American	1875	7.0		
Other Hispanic	417	6.8		
Non-Hispanic White	3965	72.9		
Non-Hispanic Black	1333	9.3		
Other race/multi-racial	236	4.0		
Sex				
Female	4056	51.4		
Male	3770	48.6		
Education				
High school degree or above	5175	78.6		
Below high school	2639	21.4		
Poverty-income-ratio				
Above poverty level	5825	79.2		
At or below poverty level	2001	20.8		
Lifetime cigarette smoker				
No	4015	50.1		
Yes	3796	49.9		
			95% CI ^2^	
	Mean	SEM ^1^	Lower	Upper
Age (R:19–85)	46.1	0.4	45.4	46.9
Mean telomere length (T/S ratio)	1.055	0.015	1.025	1.086
Serum cotinine (ng/mL)	59.3	3.2	52.7	65.8

^1^ SEM: Standard error of mean; ^2^ CI: Confidence interval.

## Data Availability

Data analyzed for this study are from the National Health and Nutrition Examination Survey, which is a publicly available dataset. These data can be found at: https://wwwn.cdc.gov/nchs/nhanes/Default.aspx (accessed on 15 June 2022).

## Supplementary Materials

The following supporting information can be downloaded at: https://www.mdpi.com/article/10.3390/ijerph191711069/s1, Table S1: Sample sociodemographic variables by race/ethnicity (N = 7826).
