# Supplementary material for: A Time-Varying Effect Model (TVEM) of the Complex Association of Tobacco Use and Smoke Exposure on Mean Telomere Length: Differences between Racial and Ethnic Groups Assessed in the National Health and Nutrition Examination Survey
Source: Int J Environ Res Public Health. 2022 Sep 4;19(17):11069. doi: 10.3390/ijerph191711069 (PMC9518386; doi:10.3390/ijerph191711069)
Supplement: Supplementary file 1 [file ijerph-19-11069-s001.zip › ijerph-1847280-supplementary.pdf]

Supplementary Table S1. Sample sociodemographic variables by race/ethnicity (N = 7826).

	Mexican American		Other Hispanic		Non-Hispanic White		Non-Hispanic Black		Other Race/ Multiracial		Total	
	N	%	N	%	N	%	N	%	N	%	N	%
Sex												
Female	971	3.3	229	3.6	2026	37.2	698	5.1	132	2.1	4056	51.4
Male	904	3.7	188	3.2	1939	35.7	635	4.2	104	1.9	3770	48.6
Education												
High school degree or above	703	3.1	230	4.4	3262	62.0	799	6.0	181	3.1	5175	78.6
Below high school	1169	3.8	185	2.4	698	11.0	532	3.3	55	0.9	2639	21.4
Poverty-income-ratio												
Above poverty level	1229	4.7	257	4.4	3292	61.4	883	6.2	164	2.7	5825	79.2
At or below poverty level	646	2.3	160	2.4	673	11.5	450	3.1	72	1.3	2001	20.8
Lifetime cigarette smoker												
No	1066	4.1	238	3.8	1862	34.7	714	5.2	135	2.3	4015	50.1
Yes	805	2.9	178	3.0	2098	38.2	617	4.1	98	1.7	3796	49.9
